# Infratentorial immature teratoma of congenital origin can be associated with a 20-year survival outcome: a case report and review of literature

**DOI:** 10.1186/s12957-019-1564-1

**Published:** 2019-01-19

**Authors:** Yazid Maghrabi, Maher E. Kurdi, Saleh S. Baeesa

**Affiliations:** 1 Division of Neurological Surgery, Department of Surgery, King Abdulaziz University Hospital, P.O. Box 80215, Jeddah, 21589 Kingdom of Saudi Arabia; 2 Department of Pathology, King Abdulaziz University Hospital, P.O. Box 9946, Jeddah, 21423 Kingdom of Saudi Arabia

**Keywords:** Brain neoplasms, Congenital, Parinaud’s syndrome, Teratoma, Posterior fossa

## Abstract

**Background:**

Congenital intracranial tumors are very rare and account for less than 2% of all childhood brain tumors. Teratomas constitute about one third to one half of these, predominantly located midline in the supratentorial region. Posterior fossa location rarely occurs and, based on the cases reported in the literature, commonly has a poor prognosis.

**Case presentation:**

A newborn female, diagnosed prenatally with hydrocephalus, is presented at birth with increasing head circumference and Parinaud’s syndrome. Magnetic resonance imaging scans demonstrated a huge posterior fossa tumor with obstructive hydrocephalus. At surgery, through a suboccipital craniotomy, complete excision was achieved of a histological-proven immature teratoma. The infant received adjuvant chemotherapy for 1 year. She had normal neurological development and remained tumor-free through her 20-year follow-up.

**Conclusion:**

The authors report this rare case of congenital posterior fossa teratoma with long-term outcome, and the literature is reviewed.

## Background

Congenital intracranial tumors are a rare occurrence, accounting for only 0.5–1.5% of all childhood brain tumors [1]. The most common of these tumors are teratomas, which comprise between 28.8 and 50.0% of central nervous system tumors [2, 3]. Teratomas are considered to be a subtype of germ cell tumors, containing all types of embryonic germ cell layers (ectoderm, mesoderm, and endoderm) [4]. Commonly, they are located in the supratentorial region, and in Wakai et al.’s review of 200 cases, only one was found in the posterior fossa [5]. Thus, infratentorial teratomas are rare entities. Histopathologically, teratomas are categorized as mature or immature [6]. Immature teratomas are associated with poor prognosis, due to the malignant behavior and presentation at a young age [6].

The Authors report this rare case of a posterior fossa congenital teratoma presented with Parinaud’s syndrome with long-term survival and review the literature.

## Case report

A newborn female of an uneventful pregnancy of a 40-year-old woman was delivered on 1 January 1999 via cesarean section (CS) at full term, because of previous CS. She is the sixth child of non-consanguineous Saudi Arabian parents who were originally from Gizan. During her gestational period, routine fetal ultrasonographic (US) scans at the 34th week reported mild ventriculomegaly without mention of any associated brain tumor.

At birth, her Apgar score was 8 and 10, and her weight was 3150 g. Her head circumference after birth was in the 90th percentile, and the anterior fontanel was 20 × 20 mm and soft. The initial neurological exam was normal, apart from a squint, and her parents were reassured.

At the age of 3 days, her mother brought her to a polyclinic because of poor feeding. She was reassured, and a change of milk formula was satisfactory for 1 week. Because of her recurrent vomiting and irritability, a computed tomography (CT) scan was performed, which reported a large posterior fossa tumor with obstructive hydrocephalus.

**Fig. 1:**
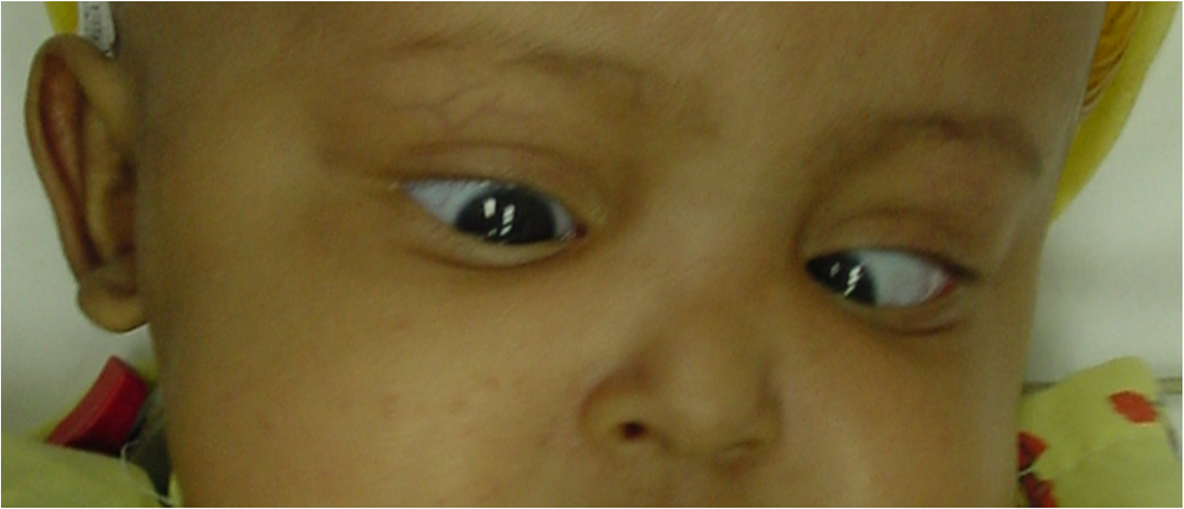
Clinical photograph of the newborn demonstrating Parinaud’s syndrome

**Fig. 2:**
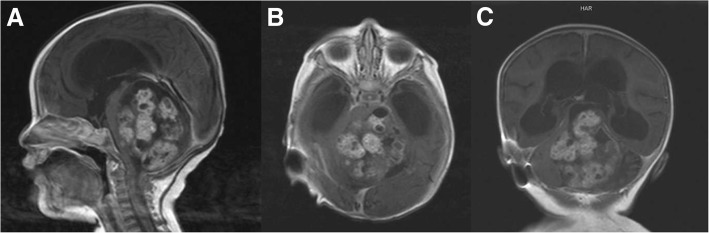
Preoperative T1-weighted **a** sagittal, **b** axial, and **c** coronal MRI scans demonstrating a 70 × 60 × 55 mm heterogeneously enhancing posterior fossa tumor with marked brain stem compression and obstructive hydrocephalus

On admission at 20 days old to King Abdulaziz University Hospital in Jeddah, she presented with frequent vomiting, poor feeding, and increasing head circumference. The general physical exam revealed an irritable and emaciated baby in the second percentile of weight for her age. Head circumference was 45 cm with visibly dilated scalp veins and bulging anterior fontanel. Neurological exam demonstrated a conscious baby with spontaneous movement of her upper and lower extremities with mild spasticity. Cranial nerves exam was uneventful apart from Parinaud’s syndrome (Fig. 1). Pupillary reflex was sluggish to light and vision was normal with no papilledema detected. Routine laboratory screening tests were within normal limits. Magnetic resonance imaging (MRI) scans revealed a heterogeneous 60 × 55 × 45 mm midline tumor filling most of the posterior fossa, causing anterior displacement of the brain stem and marked obstructive hydrocephalus (Fig. 2). The tumor was iso- to hyperintense on T1-weighted images and hypointense on T2-weighted images with heterogeneous enhancement.

**Fig. 3:**
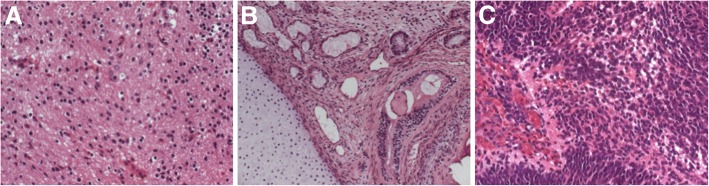
Histopathologic photomicrographs of the specimen demonstrating a predominant mature component (**a**) with a differentiated mature tissue component, including cystic respiratory and enteric epithelium and cartilage (**b**). The immature part consisted of primitive neuroblast-like cells forming a neuroepithelial rosette embedded in a hypercellular stroma (**c**). (Original magnification, × 100; hematoxylin-eosin stain)

Through a midline suboccipital craniotomy, complete resection of the tumor was achieved. During surgery, an encapsulated solid vascular tumor occupying the fourth ventricle was encountered with diverse areas of soft and firm consistency. The baby remained stable hemodynamically throughout the surgery and received a total of 250 ccs packed RBC blood. During the operation, cerebrospinal fluid (CSF) was obtained for analysis, which was within normal limits for chemistry and cell counts. CSF cytology and tumor markers studies, human chorionic gonadotropin, and alpha-fetoprotein were negative. Histopathological examination of the specimen demonstrated a variety of tissues from all three germ layers, including immature as well as mature elements. The predominant tissue was neuroectodermal, in the form of neuroepithelial rosettes and tubules, resembling neuroblastoma. Mesodermally derived immature cartilage and primitive stroma were also seen, and endodermally derived respiratory and enteric epithelium was present in the form of cystic structures (Fig. 3). The diagnosis was consistent with immature teratoma. During the postoperative period, the baby developed pneumonia that was resolved with intravenous antibiotics, and early postoperative MRI scans confirmed complete resection. She was discharged home on the 15th postoperative day in good condition. The infant received and well tolerated adjuvant carboplatin and etoposide (CARE) chemotherapy.

**Fig. 4:**
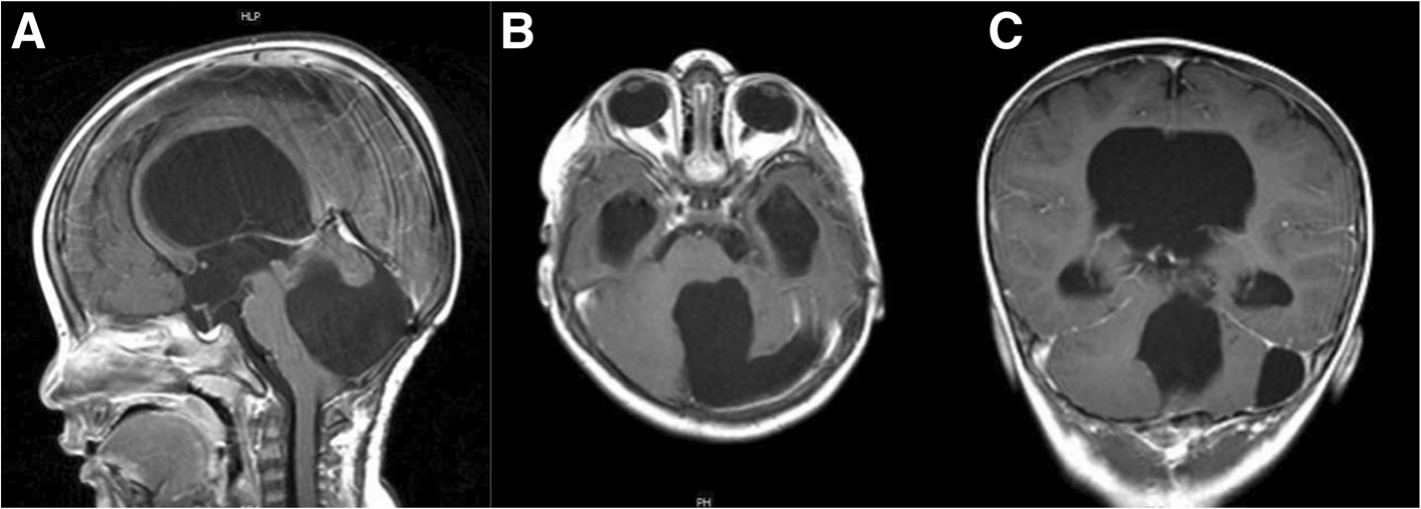
Follow-up at 12-month T1-weighted **a** sagittal, **b** axial, and **c** coronal MRI scans demonstrating no tumor recurrence

**Fig. 5:**
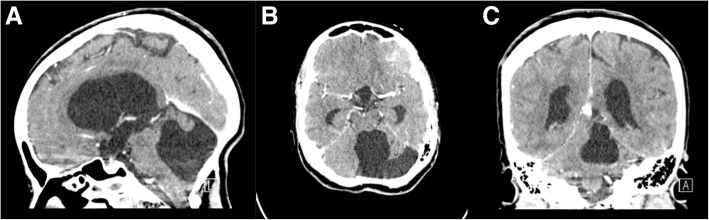
Follow-up at 20 years of age **a** sagittal, **b** axial, and **c** coronal CT scan demonstrating no tumor recurrence

At the first year anniversary follow-up, she had a mild developmental delay with a slight improvement of Parinaud’s syndrome. Follow-up MRI scans at the fifth year of age revealed the stable size of the ventricles with no tumor recurrence (Fig. 4). The patient was followed annually and had normal cognitive and neurological development; she is a university student and her recent CT scan at the age of 20 years revealed no recurrence (Fig. 5).

## Discussion

Primary brain tumors form 20% of pediatric neoplasms and are the second most prevalent cancer of childhood, after leukemia [7]. Teratomas, although tumors of gonads, also arise from the central nervous system [8]. Extragonadal teratomas are congenital midline tumors, located both intracranially and extracranially, e.g., in the mediastinum and retroperitoneal area [8]. They are considered to originate from primordial germ cells that fail to migrate properly during the initial weeks of embryonic development [8].

The incidence of intracranial teratoma is 0.5% of all brain tumors and 2 to 4% of intracranial tumors in the pediatric age group [9–11]. This becomes statistically more significant for intracranial tumors presenting at or around birth. Congenital teratomas are the most frequent intracranial tumor of the perinatal and neonatal period and are five times more common than astrocytomas, which are next in frequency at this age [3]. That is why they are considered as the leading prenatal neoplasms, according to multiple reviews [3]. Considering tumors in the pediatric age group, male children are affected more than female children, but in neonates, females predominate [9]. Both supra- and infratentorial tumors have been reported with the majority being supratentorial, contrary to the usual pattern of brain tumors observed in the pediatric age group [12].

Common presenting symptoms and signs of such tumors include symptoms related to the outflow obstruction of cerebrospinal fluid resulting in a headache, vomiting, and papilledema (Table 1) [4, 13]. Moreover, the progressive increase in head size has also been observed in many reported cases including our current case (Table 1) [4, 13–16]. Infants with teratoma present with seizures in around 20% of cases [4, 13]. Compression of the tectal plate can result in what is known as Parinaud’s syndrome, which is characterized by vertical gaze palsy, convergence palsy, and accommodation palsy [14]. The tectal plate can be compressed through several mechanisms including hydrocephalus, causing either (a) direct compression of the tectal plate of the midbrain by the distended ventricular system or (b) secondary herniation, with resultant brainstem ischemia or neuronal distortion.

**Table 1:** Review of reported congenial teratoma cases confined to the posterior fossa

Author (year)	Mode of delivery and Apgar score	Age at diagnosis	Signs and symptoms	Tumor size	Tumor location	Histopathology	Neurosurgical intervention	Outcome	Follow-up
Current case	C section, 8 at 1 min, 10 at 5 min	20 days	Vomiting, poor feeding, and increasing head circumference	6 × 5.5 × 4.5 cm	Posterior fossa	Immature teratoma	Total resection	Alive	20 years
Algahtani et al. (2018) [22]	SVD, NR	5 years	Headache, vomiting, and irritability	NR	Posterior fossa	Mature teratoma	Total resection	Alive	6 years
Daugherty et al. (2016) [23]	NR	15 years	Headache, imbalance, and papilledema	NR	Posterior fossa	Mature teratoma	Total resection	Alive	9 months
Ferraz et al. (2014) [24]	NR	2 months	Down’s syndrome	NR	Posterior fossa	Mature teratoma	Incomplete resection	Recurrence	2 years
Thust et al. (2014) [14]	C-section, NR	11 weeks	Crying, irritability, and macrocephaly	NR	Posterior fossa	Teratoma	Incomplete resection	Recurrence	18 months
Fukuoka et al. (2014) [15]	SVD, 9 at 1 min, 10 at 5 min	12 days	Vomiting, head circumference enlargement	NR	Posterior fossa	Immature teratoma	Total resection	Alive	3 years
Algahtani et al. (2013) [4]	SVD, NR	5 years	Headache, vomiting, and irritability	NR	Posterior fossa	Mature teratoma	Total resection	Alive	1 year
Gao et al. (2013) [13]	NR, 9	4 months	Seizures, downward gaze palsy, binocular convergence	NR	Posterior fossa	Immature teratoma	Total resection	Alive	18 months
Rios et al. (2013) [25]	C section, NR	Prenatal	Incidental finding	5.3 × 4.8 cm	Posterior fossa	Immature teratoma	Biopsy	Dead	NA
Bakhtiar et al. (2012) [16]	SVD, NR	2 months	Lethargy and progressive head enlargement	5 cm	Posterior fossa	Immature teratoma	Total resection	Recurrence	4 years
Das et al. (2007) [26]	NR, NR	2 years	Headache vomiting, imbalance, and speech disturbance	NR	Posterior fossa	Immature teratoma	Incomplete resection	Dead	NA
Desai et al. (2001) [6]	C section, NR	10 days	Rapid head enlargement, vomiting, irritability	NR	Posterior fossa	Immature teratoma	Total resection	Dead	NA
Bavbek et al. (1999) [27]	SVD, NR	5 years	Head enlargement	NR	Posterior fossa	Mature teratoma	Total resection	Alive	NR
Hayashi et al. (1984) [28]	NR	27 days	NR	NR	Posterior fossa	Immature teratoma	Total resection	Dead	NA

*SVD* spontaneous vaginal delivery, *NR* not reported, *C section* caesarian section, *NA* not applicable

Furthermore, structural compression of the tectal plate by the tumor generates abnormal forces resulting in either brainstem ischemia or neuronal distortion before the development of hydrocephalus. Abnormal development of the tectal plate nuclei can be another cause of such syndrome in this patient population. This can be illustrated in our current case: partial improvement of Parinaud’s syndrome resulted after successful decompression, and no hemorrhage or infarction within the brainstem in the postoperative MRI scans suggests that tectal plate neurons may have been incompletely developed.

In a patient with congenital cerebellar teratoma, diagnosis may be prenatal or postnatal [17]. Prenatal diagnosis has increased compared to the past due to better prenatal follow-up and excellent radiological workups [17]. Diagnosis can be suspected on routine fetal ultrasound examination [17]. It may show intrauterine hydrocephalus, enlarged biparietal diameter, or brain tumor [17]. Antenatal diagnosis is usually possible as early as the 20th week of gestation [17, 18]. In addition to hydrocephalus, other common clinical conditions at the time of presentation are polyhydramnios, followed by respiratory distress and stillbirth [12]. If fetal ultrasound is suggestive of hydrocephalus due to a tumor, MRI of the fetus must be advised for definitive localization of the size and extent of the tumor and hydrocephalus [17, 18]. Usually, head circumference is enlarged before birth, and cephalopelvic disproportion becomes an indication for caesarian section [17–19]. After delivery, parents may bring the baby in with a history of progressively increasing head circumference. Other history and examination are suggestive of hydrocephalus, which at this age is usually congenital [4, 15, 16]. Magnetic resonance imaging of the brain with and without gadolinium in such patients is diagnostic for tumor [4]. It usually shows a solid and cystic mass with heterogeneous enhancement and elements of calcification [4]. There is often hydrocephalus in posterior fossa teratomas as in our case report. Tumor markers like alpha-fetoprotein and β HCG help to suspect a malignant component in teratomas preoperatively [4].

Once the diagnosis of posterior fossa tumor is confirmed, the treatment of choice is complete excision of the lesion [4]. Afterward, complete clinical, as well as radiological, follow-up is recommended.

Poor survival of infants with congenital intracranial teratoma has been shown; a large number die prenatally and the rest shortly after birth [2]. In their literature review, Wakai and his colleagues found the 1-year survival rate of infants with congenital intracranial teratoma reaching 7.2% [5]. However, there have been some reports in which infants survived for a long time, such as our current case and that of Fukuoka et al. [15]. Long-term survival can be attributed to complete resection, yet this has not been entirely proven [20]. The location and the size of the teratoma are considered as significant prognostic factors rather than the histological grade [2]. Thus, posterior fossa teratomas are associated with poor outcome. Kitahara et al. have demonstrated that the use of neoadjuvant chemotherapy CARE can decrease tumor size and vascularity, paving the way for achieving total tumor resection and resulting in lower morbidity and mortality [21]. In their report, they unsuccessfully attempted surgical resection before initiation of neoadjuvant chemotherapy and then succeeded in achieving near-total resection after completing the CARE regimen [21]. This was unlike our current case, in which adjuvant chemotherapy was used after achieving total tumor resection. The unique aspect of our case is that it is the only one reporting a patient surviving more than 20 years after undergoing complete excision of a posterior fossa immature teratoma.

## Conclusion

Congenital posterior fossa teratoma is a rare tumor that presents with significant large size and massive obstructive hydrocephalus. Early surgical resection is recommended with adequate perioperative care. Regular clinical and radiological follow-up is essential to assess the child’s neurological function and watch for early recurrence. Despite insufficient data in the literature about factors that favor a good outcome for posterior teratoma, we note that complete surgical resection is a significant prognosticator.

## Abbreviation

CARE: Carboplatin and etoposide
